# Data on the prevalence and distribution of carbapenemase genes in Enterobacterales species isolated from clinical specimens in the center of Irans

**DOI:** 10.1016/j.dib.2021.107368

**Published:** 2021-09-13

**Authors:** Farzad Badmasti, Omid Azizi, Mohammad Ali Mohaghegh, Hamid Solgi

**Affiliations:** a Department of Bacteriology, Pasteur Institute of Iran, Tehran, Iran; b Microbiology Research Center (MRC), Pasteur Institute of Iran, Tehran, Iran; c Health Sciences Research Center, Torbat Heydariyeh University of Medical Sciences, Torbat Heydariyeh, Iran; d Department of Laboratory Sciences, School of Paramedical Sciences, Torbat Heydariyeh University of Medical sciences, Torbat Heydariyeh, Iran; e Department of Laboratory Medicine, Amin Hospital, Isfahan University of Medical Sciences, Isfahan, Iran

**Keywords:** Enterobacterales species, Antibiotic resistance, Carbapenemase genes

## Abstract

Carbapenem resistance in Enterobacterales is a major and persistent public health problem worldwide. In current research, we present data of 96 Enterobacterales species collected from a clinical hospital in Isfahan, Iran. The bacterial identification was performed by standard biochemical tests and API 20E methods. Agar disk diffusion assay was performed to determine the phenotypic antibiotic resistance of strains. Polymerase chain reaction (PCR) was carried out to detect carbapenemase genes. In this manuscript, multiple antimicrobial resistance phenotype such as multiple carbapenem resistance determinants were detected. The data would provide important information on distribution of carbapenemase genes of those pathogenic bacteria in Iran.

**Specifications Table**

**Table:** 

Subject	Microbiology: Bacteriology
Specific subject area	Antimicrobial resistance
Type of data	Table and Figure
How data were acquired	Culture methods, PCR assay
Data format	Raw and Analyzed
Parameters for data collection	Carbapenem resistant Enterobacterales species were identified in CHROMagar KPC medium. The diameter of inhibition zones on agar was measured and interpreted as resistant by referring to breakpoints suggested by clinical and laboratory standards institute (CLSI). The data analyses of carbapenemase genes were conducted using bioinformatics tools.
Data source location	Al-Zahra Hospital, Isfahan University of Medical Sciences, Isfahan, Iran.
Data accessibility	All data are presented in this article.
Related research article	H. Solgi, F. Badmasti, C.G. Giske et al., Molecular epidemiology of NDM-1- and OXA-48-producing *Klebsiella pneumoniae* in an Iranian hospital: clonal dissemination of ST11 and ST893, J Antimicrob Chemother, 73 (2018) 1517–1524 [1].

**Value of the Data**

• The dataset will provide important relevant information on Enterobacterales species that pose a major public health threat due to their involvement in the spread of antibiotic resistance.
• The data will aid in the discussion of the possible spread of carbapenemase genes among Enterobacterales species.
• These data provide insights into antibiotic resistance in Enterobacterales species that will be useful to patients and clinicians.
• Data on the prevalence of carbapenemase-producing Enterobacterales will be useful for further infection control and stewardship surveillance programs.

## 1 Data Description

**Fig. 1:**
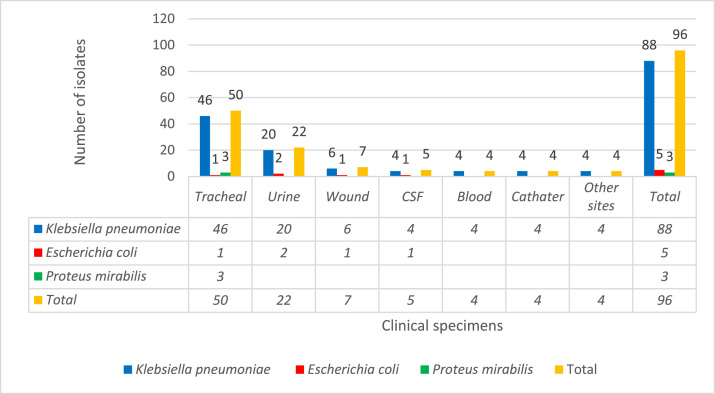
Frequency distribution of carbapenem resistant Enterobacterales species (*K. pneumoniae, E. coli*, and *P. mirabilis*) based on clinical specimens in our hospital.

**Fig. 2:**
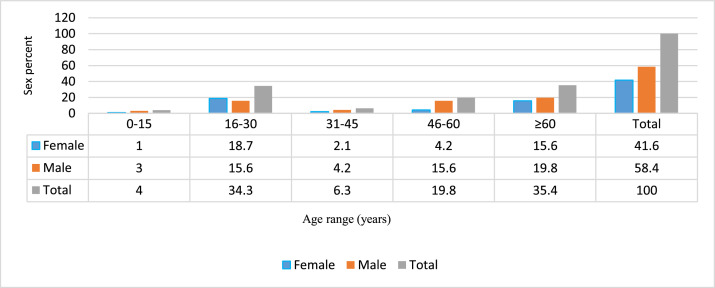
Frequency of carbapenem resistant Enterobacterales species in relation to patient's age.

**Fig. 3:**
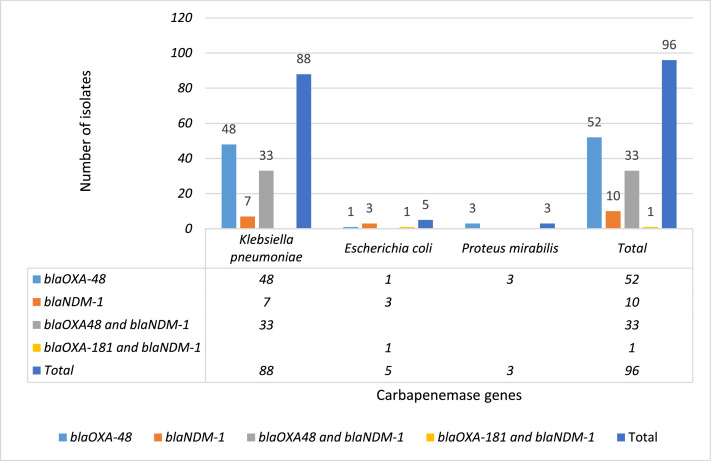
The distribution of carbapenemase genes among clinical carbapenem-resistant Enterobacterales species (*K. pneumoniae, E. coli*, and *P. mirabilis*) isolated in clinical specimens.

The prevalence of carbapenem-resistant Enterobacterales (CRE) bacteria is on the rise worldwide, especially in Iran and is a worrying public health threat [1], [2], [3], [4], [5]. The data presented show the frequency distribution of carbapenem-resistant Enterobacterales by basis on clinical specimens and age of patients collected from clinical specimens at Al-Zahra teaching hospital (Figs. 1 and 2). Al-Zahra teaching hospital with about 650 beds is located in the south of Isfahan in the center of Iran. Table 1 lists the antimicrobial susceptibility testing of extensively drug resistant (XDR). In addition, the carbapenemase genes present in the genome of CRE are shown in Fig. 3. Raw data is provided in the Microsoft Excel Worksheet in the supplementary data.

**Table 1:** Antibiotic susceptibility testing of carbapenem resistant Enterobacterales species isolated in clinical specimens from a teaching hospital in central Iran.

Species	Antimicrobial resistance phenotype	XDR	No. of isolates
*Klebsiella pneumoniae*	MEM-IMI-CAZ-CTX-FEP-AMK-GEN-CIP-FOF-CST	+	2
	MEM-IMI-CAZ-CTX-FEP-CIP	-	8
	MEM-IMI-CAZ-CTX-FEP-AMK-GEN-CIP	+	41
	MEM-IMI-CAZ-CTX-FEP-GEN-CIP	+	27
	MEM-IMI-CAZ-CTX-FEP-GEN-CIP-FOF	+	1
	MEM-IMI-CAZ-CTX-FEP-GEN-CIP-FOF-CST	+	1
	MEM-IMI-CAZ-CTX-FEP-CIP-FOF	+	1
	MEM-IMI-CAZ-CTX-FEP-CIP-CST	+	4
	MEM-IMI-CAZ-CTX-FEP-AMK-GEN-CIP-FOF	+	3
*Escherichia coli*	MEM-IMI-CAZ-CTX-FEP-CIP,FM	+	3
	MEM-IMI-CAZ-CTX-FEP-AMK-CIP	+	2
*Proteus mirabilis*	MEM-IMI-CAZ-CTX-FEP-AMK-GEN-CIP	+	2
	MEM-IMI-CAZ-CTX-FEP-AMK-CIP	+	1

*Abbreviations:* MEM, meropenem; IMI, imipenem; CAZ, ceftazidime; CTX, cefotaxime; FEP, cefepime; AMK, amikacin; GEN, gentamicin; CIP, ciprofloxacin; FOF, fosfomycin; CST, colistin, FM, nitrofurantoin; XDR, extensively drug-resistant.

## 2 Experimental Design, Materials and Methods

### 2.1 Bacterial isolates and agar disk diffusion assay

The Enterobacterales were collected from a total of 423 clinical specimens such as ascites, blood, wound, catheters, cerebrospinal fluid (CSF), tracheal and urine from hospitalized patients and outpatients in different wards of an educational hospital affiliated to Isfahan University of Medical Sciences, Isfahan, Iran. The clinical samples were cultured on blood agar and Mac-Conkey agar (Merck) and bacteria isolated and identified using standard biochemical tests and API 20E (bioMérieux, Marcy-l'Etoile, France). Triple Sugar Iron (TSI), SIM (Sulfide, Indole, Motility), Simmons citrate, Methyl Red & Vogues-Proskauer (MR-VP) and urease can be mentioned as the biochemical tests used for identifying the Enterobacterales species. In addition, to detect CRE strains, all samples were subcultured directly on CHROMagar KPC medium (CHROMagar Company, Paris, France). CRE colonies on CHROMagar KPC plates were identified according to the manufacturer's instructions. Agar disk diffusion assay was performed using commercially available antibiotic plates (MAST, UK) on Mueller-Hinton agar according to the guidelines of clinical and laboratory standards institute (CLSI) and European Committee on Antimicrobial Susceptibility Testing (EUCAST) [6,7]. The following antibiotics were tested: imipenem (IMP: 10 µg), meropenem (MEM: 10 µg), ceftazidime (CAZ: 30 µg), cefotaxime (CTX: 30 µg), cefepime (FEP: 30 µg), ciprofloxacin (CIP: 5 µg), amikacin (AMK: 30 µg), gentamicin (GEN: 30 µg), and fosfomycin (FOF: 200 µg) (Mast Group Ltd., Merseyside, United Kingdom). The diameter of inhibition zones was measured and interpreted as resistant by referring to breakpoints suggested by CLSI. The minimal inhibitory concentration (MIC) of colistin were determined by broth macrodilution method using colistin sulfate (Sigma-Aldrich), and EUCAST breakpoints were used for interpretation. *K. pneumoniae* ATCC700603 and *Escherichia coli* ATCC 25922 was used as quality control strain for antimicrobial susceptibility testing. The data entry and analysis were done by using the WHONET 5.6 software. Strains that were resistant to all antimicrobials except two or less antimicrobial categories were considered as XDR.

### 2.2 Detection of carbapenemase genes using PCR

Genomic DNA was extracted using the DNA genomic extraction kit (Thermo scientific, Lithuania). Polymerase chain reaction (PCR) were carried out using primers specific for the genes encoding *bla*_KPC_, *bla*_GES_ *bla*_OXA-48_, *bla*_NDM_, *bla*_VIM_ and *bla*_IMP_ as previously described [8]. The PCR amplicons were sequenced using an ABI Capillary System (Macrogen Research, Seoul, Korea), and the DNA sequence of each gene was compared to the sequences in the GenBank nucleotide database at http://www.ncbi.nlm.nih.gov/blast/. The sequences were then manually assembled by using CLC main workbench software version 5.5 (CLC Bio, Aarhus, Denmark).

## Ethics Statement

The study was approved by the research and the Ethics Committee of the Pasteur Institute of Iran (project No: IR.PII.REC.1395.51). No ethical approval was obtained for using the clinical isolates since they were collected during the routine diagnostic laboratory at our hospital.

## CRediT Author Statement

**Farzad Badmasti:** Investigation, Formal analysis; **Omid Azizi:** Writing – original draft, Conceptualization, Investigation, Formal analysis, Writing – review & editing; **Hamid Solgi:** Writing – original draft, Formal analysis, Conceptualization, Investigation, Writing – review & editing.

## Declaration of Competing Interest

The authors declare that they have no known competing financial interests or personal relationships which have, or could be perceived to have, influenced the work reported in this article.

## Appendix Supplementary Materials

Image, application 1
